# Mesenchymal stem cell-derived extracellular vesicles ameliorate heat stroke-induced hippocampal injury and modulate VIM-AS1-associated miR-34a-5p/Per2 signaling

**DOI:** 10.3389/fimmu.2026.1873819

**Published:** 2026-09-14

**Authors:** Lu Wang, Min Wang, Yuyan Liu, Yang Bai, Yun Li, Yuan Cao, Chengjin Wang, Zhen Gao, Yiqi Wu, Zihui Deng, Hongjun Kang

**Affiliations:** 1 Department of Critical Care Medicine, the Fifth Medical Centre, Chinese PLA General Hospital, Beijing, China; 2 National Key Laboratory of Kidney Diseases, Beijing Key Laboratory of Kidney Disease Research, National Clinical Research Center for Kidney Diseases, Beijing, China; 3 Medical School of Chinese PLA, Beijing, China; 4 Department of Critical Care Medicine, the First Medical Centre, Chinese PLA General Hospital, Beijing, China; 5 Department of Critical Care Medicine, Beijing Armed Police Corps Hospital, Beijing, China; 6 Department of Emergency Medicine, the First Medical Centre, Chinese PLA General Hospital, Beijing, China; 7 Department of Critical Care Medicine, PLA General Hospital of Southern Theatre Command, Guangzhou, China; 8 Department of Basic Medicine, Medical School of Chinese PLA, Beijing, China

**Keywords:** central nervous system, exosomes, heat stroke, mesenchymal stem cells, microglial cell

## Abstract

**Background:**

Central nervous system (CNS) injury is a major determinant of mortality and persistent disability after heat stroke. Mesenchymal stromal/stem cell-derived extracellular vesicles (MSC-EVs) are candidate cell-free immunomodulatory therapeutics, but their effects and molecular correlates in heat stroke-associated brain injury remain incompletely defined. We investigated whether MSC-EVs attenuate hippocampal inflammation and alter microglial phenotype-associated markers after heat stroke.

**Methods:**

Human umbilical cord MSC-EVs were isolated by differential ultracentrifugation and characterized by transmission electron microscopy, nanoparticle tracking analysis, and immunoblotting for EV-enriched and non-EV markers. In a mouse heat stroke model, MSC-EVs (200 μg total protein per mouse) were administered intravenously. Neurological outcomes, hippocampal pathology, inflammatory mediators, IBA1 immunoreactivity, and phenotype-associated markers were evaluated. BV2 heat-stress and MSC co-culture experiments were used to examine VIM-AS1-associated miR-34a-5p/Per2 signaling.

**Results:**

MSC-EV treatment improved survival and neurological scores and reduced hippocampal injury and inflammatory readouts after heat stroke. In mouse brain and BV2 models, treatment was associated with lower expression of inflammatory-associated markers and higher expression of repair-associated markers. EV RNA sequencing identified abundant VIM-AS1. VIM-AS1 gain- and loss-of-function experiments altered miR-34a-5p and Per2 expression, while miR-34a-5p directly repressed a conserved mouse Per2 3′UTR reporter. VIM-AS1 overexpression also improved inflammatory and neurological readouts *in vivo*.

**Conclusions:**

MSC-EVs attenuated heat stroke-induced hippocampal injury and were associated with coordinated changes in VIM-AS1, miR-34a-5p, and Per2. These findings support MSC-EVs and VIM-AS1-associated signaling as candidates for further mechanistic investigation, while not establishing VIM-AS1 as the sole or dominant active EV cargo.

## Highlights

- Human umbilical cord MSC-EVs improved survival and neurological and hippocampal outcomes in a mouse heat stroke model.
- MSC-EV treatment shifted a panel of inflammatory- and repair-associated microglial markers and reduced neuroinflammatory mediators.
- VIM-AS1 gain-of-function was associated with reciprocal miR-34a-5p/Per2 changes, but VIM-AS1 is interpreted as one candidate contributor among multiple EV cargoes.

## 1 Introduction

Heat stroke (HS) is a life-threatening heat illness defined by marked hyperthermia accompanied by central nervous system dysfunction (1). As anthropogenic warming increases the frequency and intensity of extreme-heat events, heat-related morbidity and mortality are rising across regions (2–4). Neurological injury is not restricted to the acute encephalopathic phase: in a recent six-hospital cohort of 206 patients with HS, 44 patients (21.4%) had neurological sequelae at discharge (5), consistent with our earlier cohort in which 24.4% developed longer-term neurological sequelae (6). Severe CNS injury and multiple organ dysfunction therefore remain major determinants of death and disability, yet treatment is still largely supportive.

Neuroinflammation is a central component of HS-induced CNS injury (7). Neuroimaging may reveal cortical, hippocampal, cerebellar, and subcortical abnormalities (8), and experimental heat strain produces temporally dynamic cytokine responses in brain tissue (9). Microglia are resident CNS macrophages that adopt heterogeneous, context-dependent transcriptional and functional states rather than discrete binary phenotypes (10–13). Our previous work showed increased hippocampal IBA1 immunoreactivity and inflammatory mediators after HS (14). The present study therefore evaluates a panel of inflammatory- and repair-associated markers together with IBA1 morphometry, while avoiding the assumption that any single marker defines microglial activation.

MSCs have immunomodulatory, anti-apoptotic, and tissue-repair properties (15). A substantial part of their paracrine activity is mediated by secreted extracellular vesicles, which carry proteins, lipids, and nucleic acids and can alter recipient-cell signaling. MSC-EVs have reduced neuroinflammation in experimental cerebral ischemia and other acute CNS injuries (16–19). In parallel, induced-pluripotent-stem-cell strategies are being explored for ischemic stroke, illustrating broader interest in regenerative and cell-derived interventions for neurological injury while also highlighting unresolved safety and standardization challenges (20). Because EVs are non-replicating and can be standardized as a cell-free product, they may mitigate some risks associated with viable-cell therapy. However, EV preparations are heterogeneous, their active cargo is rarely attributable to a single molecule, and rigorous characterization is required under current MISEV recommendations (21).

Our previous studies showed that viable MSC administration reduced HS-associated neuroinflammation, improved neurological deficit scores, and increased survival (14, 22, 23). The present work extends those studies in three ways: it tests a cell-free MSC-EV preparation, examines microglial phenotype-associated readouts *in vivo* and in a BV2 heat-stress model, and screens EV-enriched long non-coding RNAs for candidate signaling mechanisms. We focused on VIM-AS1 and evaluated its association with miR-34a-5p and Per2. Because some experiments used MSC co-culture or AAV-mediated VIM-AS1 expression rather than purified VIM-AS1-modified EVs, we interpret this pathway as a candidate or partial mechanism rather than the exclusive mediator of MSC-EV protection.

## 2 Materials and methods

### 2.1 Animals and experimental design

Eight-week-old male C57BL/6J mice were purchased from Huafukang Bioscience Co., Ltd. (Beijing, China) and housed at the Experimental Animal Center of the Chinese PLA General Hospital under controlled conditions (20 ± 2 °C; 50 ± 5% relative humidity; food and water ad libitum). Mice were randomized before intervention to control, HS, HS plus MSC-EV, AAV-vector, or AAV-VIM-AS1 groups and the prespecified collection times. Endpoint-specific biological sample sizes are reported in the Methods and figure legends. Survival was evaluated in a separate cohort of 30 mice (n = 15/group for HS and HS plus MSC-EV); these animals were not used for tissue, molecular, histological, or neurological-score endpoints. Neurological scores were repeatedly measured in the same mice (n = 5/group). The assessor performing neurological scoring was unaware of group allocation. No prospective power calculation was performed; sample sizes were based on prior HS experiments and the exploratory design.

For VIM-AS1 gain-of-function, mice received 100 μL AAV9 carrying human VIM-AS1 (GPAAV-CMV-H_VIM-AS1-EF1-ZsGreen1-WPRE; 1.15 × 10^12 vector genomes [vg]/mL; 1.15 × 10^11 vg/mouse; Genomeditech, Shanghai, China) by tail-vein injection. Vector controls received the corresponding empty AAV9. HS was induced 7 days later. At the experimental endpoint, mice were anesthetized with intraperitoneal sodium pentobarbital (60 mg/kg) and euthanized by cervical dislocation. Procedures were approved by the Experimental Animal Welfare Ethics Committee of the Chinese PLA General Hospital (No. 2022-X18-19; approval date, March 14, 2022) and followed institutional animal-care guidance.

### 2.2 Establishment of the HS model and treatment protocol

The HS model was established as previously described (14). Briefly, after chloral hydrate anesthesia, mice were placed in a climate chamber with temperature increased from 25 °C to 39.5 °C over 1 h (0.25 °C/min) at 60% humidity. Rectal temperature was monitored every 10 min, and HS was defined when it reached 42.7 °C. Mice were then returned to room temperature and given 0.3 mL saline intraperitoneally.

MSC-EV-treated mice received 200 μg EV protein per mouse by tail-vein injection immediately after removal from the heat chamber; HS vehicle controls received an equal volume of saline. Control mice were not exposed to the HS protocol. Particle-equivalent dosing was not available for the original intervention experiment.

### 2.3 Cell culture

BV2 mouse microglial cells (National Biomedical Experimental Cell Resource Bank, 1101MOU-PUMC000063; RRID: CVCL_XD66; obtained July 23, 2021) were mycoplasma-free and maintained in DMEM (Gibco) containing 10% FBS at 37 °C and 5% CO_2_. The *in vitro* heat-stress condition was prespecified from our established model: cells were incubated at 42 °C for 2 h and recovered at 37 °C for 6 h before analysis. This reductionist exposure models cellular heat stress and is not intended to reproduce the systemic physiology of clinical heat stroke.

### 2.4 Transwell assays

A 0.4-μm Transwell system (Corning, USA) was used to examine indirect communication between HUCMSCs in the upper compartment and BV2 cells in the lower compartment. Cells were cultured in DMEM containing 10% FBS at 37 °C and 5% CO_2_. This design permits exchange of EVs and soluble factors but does not isolate EV-specific effects or exclude free extracellular RNA; it is therefore described as an indirect co-culture assay rather than proof of exosomal transfer.

### 2.5 Isolation and characterization of MSC-EVs

HUCMSCs were isolated and expanded as previously described (14). Umbilical cords were washed in PBS, vessels were removed, and approximately 1-mm³ tissue fragments were cultured in DMEM/F12 with 10% FBS at 37 °C and 5% CO_2_. Medium was changed every 2 days and cells were passaged at approximately 80% confluence. Passage-5 HUCMSCs were used. Before EV collection, cells were cultured for 48 h in medium containing EV-depleted FBS.

Conditioned medium was centrifuged at 300 × g for 10 min and 3,000 × g for 20 min, filtered through a 0.22-μm membrane, concentrated by ultrafiltration at 4,000 × g for 5 min, and ultracentrifuged at 100,000 × g for 2 h at 4 °C. The pellet was resuspended in PBS and ultracentrifuged again at 100,000 × g for 2 h; the final pellet was resuspended in 200 μL PBS and stored at −80 °C. An archived aliquot from the same production batch underwent confirmatory analytical characterization. TEM was performed after uranyl acetate negative staining; NTA was performed using a ZetaView instrument; and immunoblotting compared EV preparations with MSC lysates for CD9, CD63, CD81, TSG101, and the non-EV marker Calnexin. NTA yielded a mean diameter of 123.1 nm, a modal diameter of 104.6 nm, and 3.6 × 10^10 particles/mL. The particle-to-protein ratio was 4.99 × 10^10 particles/mg. Because no free-nucleic-acid contamination assay was performed and the preparation was not demonstrated to be a pure exosome subtype, we use the operational term MSC-EV or small-EV-enriched preparation in accordance with MISEV2023 (21).

### 2.6 Neurological deficit scoring

Neurological function was assessed before HS and at 3, 12, 24, and 72 h using a 10-item neurological severity score (24). One point was assigned for failure of each task: mono-/hemiparesis; inability to traverse 3-, 2-, or 1-cm beams; inability to balance on a 1-cm beam or 0.5-cm round rod; failure to exit a 30-cm circle within 2 min; inability to walk straight; loss of startle response; and loss of seeking behavior. Scores range from 0 to 10, with higher scores indicating greater impairment. Animals were identified by coded cage and animal numbers, and scoring was performed at fixed times by an investigator not involved in modeling, treatment, or allocation. No observation was excluded solely because the score was 0.

### 2.7 Hematoxylin-Eosin (HE) and Nissl staining

Hippocampal tissues were fixed in 4% paraformaldehyde, paraffin-embedded, and sectioned at 5 μm. For HE staining, sections were dewaxed, stained with hematoxylin and eosin, dehydrated, cleared, and mounted. For Nissl staining, sections were dewaxed and stained with 1% toluidine blue at 60 °C for 40 min, followed by dehydration, clearing, and mounting. Images were acquired using the Pannoramic MIDI CaseViewer 2.0 system (3DHISTECH Ltd.).

### 2.8 Real-time quantitative PCR (qPCR)

Total RNA was extracted from BV2 cells or dissected hippocampal tissue using TRIzol (Invitrogen) and reverse-transcribed with an M-MLV RT kit (Promega). qPCR was performed with GoTaq qPCR Master Mix (Promega). VIM-AS1, Per2, and mRNA phenotype markers were normalized to β-actin, whereas miR-34a-5p was normalized to U6. Relative expression was calculated using the 2^−ΔΔCt method. Primer sequences are listed in **Table 1**. Independent biological samples, not technical wells, were the units of statistical analysis; technical reactions were averaged before analysis. A supplementary raw-Ct audit showed no detectable group effect for β-actin in the core BV2 experiment (CV 1.39%; one-way ANOVA P = 0.1026; exact permutation P = 0.1036) or for U6 (CV 2.33%; ANOVA P = 0.3095; exact permutation P = 0.2964). A dedicated multi-reference geNorm/NormFinder experiment was not performed.

**Table 1:** Primer sequences.

Gene	Forward primer (5’-3’)	Reverse primer (5’-3’)
VIM-AS1	GAGAAGAGGCGAACGA	TCACCACGAGTCCATTA
miR-34a-5p	GATCTGGCAGTGTCTTAGCT	GTGCAGGGTCCGAGGT
Per2	TGGTGGTCAACATTCC	TCAGTGGCTGCTTTCT
TNF-α	GGTGCCTATGTCTCAGCCTCTT	GCCATAGAACTGATGAGAGGAG
IL-1β	ATAAGCCCACTCTACACCT	ATTGGCCCTGAAAGGAGAGA
IL-6	AGTTGCCTTTCTTCTGGGACT	GTGTGGTTGTTGACCTTGGA
IL-10	GGCAGATCTATGCTTGGCTCAGCACTG	GCGATATCCCTGCAGTCCAGTAGACG
TGF-β	CCTGGAGCGCAACCTGACC	GATGTCTGCTGGCGAGGTTG
MCP-1	TGGCTGTTGGTTTGTGGA	AGGAGTAGGCAATCGGTA
iNOS	AGATGGCTGTACAAGGGTTTG	AGGTGAGGTGGCTTTGTGGT
Arg-1	CCAGTCTGGCAGTTGGAAG	GGTTGTCAGGGGAGTGTTGATG
Fizz-1	CACCTCTTCACTCGAGGGACAGTTG	GGTCCCAGTGCATATGGATGAGACC
CD86	GGTGGAGGAAGGAGGAAGAT	GGGGTGGTAGAGGTGGTGTT
CD206	TGGCTGTGTGGTGGAAGGA	GTGGTGGGTGGTTGGTGT
β-actin	TGCTGTCCCTGTATGCCTCT	TTTGATGTCACGCACGATTT
U6	CTCGCTTCGGCAGCACA	AACGCTTCACGAATTTGCGT

### 2.9 Flow cytometry

Flow cytometry quantified CD86 and CD206 as phenotype-associated surface markers, not as exclusive definitions of discrete M1/M2 states. Approximately 1 × 10^5 BV2 cells were washed and stained for 30 min at room temperature with anti-CD86–FITC (Invitrogen/eBioscience, 11-0862-81) or anti-CD206–PE (Invitrogen/eBioscience, 12-2061-80), washed, and analyzed on a BD FACSCalibur using 488-nm excitation. Debris was excluded using forward- and side-scatter gates, and a consistent marker-positive gate was applied across groups using the available control files. The archived records did not document a dedicated viability dye or a full FMO panel; this limitation is stated explicitly.

### 2.10 Immunofluorescence staining

Hippocampal tissues were cryoprotected in sucrose, embedded in optimal cutting temperature compound, and sectioned at 40 μm. Sections were permeabilized with 0.5% Triton X-100, blocked with normal sheep serum, incubated overnight at 4 °C with anti-IBA1 antibody (1:100; Servicebio), and incubated with anti-rabbit IgG secondary antibody (1:100; Servicebio) for 1 h at room temperature. Nuclei were counterstained with DAPI and images were acquired with the Pannoramic MIDI/CaseViewer system (3DHISTECH). For the AAV cohort, five representative hippocampal fields from each of three mice per group were analyzed. A single global red-channel-specific threshold was applied to all images. IBA1-positive area and a skeleton-based process-complexity index (skeleton length divided by segmented IBA1 area) were calculated for each field. The five fields were averaged to obtain one value per mouse, and the animal, rather than the field, was the biological unit. This image-derived index is a morphology proxy and not a phagocytosis assay.

### 2.11 Enzyme-Linked Immunosorbent Assay (ELISA)

Supernatants from BV2 cells were collected, and TNF-α, IL-1β, IL-6, IL-10, and TGF-β levels were measured using commercial ELISA kits (Beyotime, Shanghai, China) according to the manufacturer’s instructions.

### 2.12 Western Blot (WB)

BV2 cells were lysed in RIPA buffer (Servicebio, G2002), and protein concentration was determined using a BCA kit (Servicebio, G2026). Equal amounts of protein were separated by SDS-PAGE (Servicebio, G2003), transferred to PVDF membranes (Servicebio, G6015-0.45), blocked with 5% non-fat milk in TTBS, and incubated overnight at 4 °C with primary antibodies against iNOS (Affinity, AF0199), Arg1 (Affinity, DF6657), Per2 (Affinity, DF12304), and β-actin (KANGCHENG, KC-5A08) (all 1:2000). Membranes were then incubated with secondary antibodies (1:5000) for 2 h at room temperature. β-actin served as the internal control, and band intensity was quantified using ImageJ.

### 2.13 RNA isolation and RNA-sequencing

Total RNA from the HUCMSC-EV preparation was extracted using TRIzol (Thermo Fisher Scientific, USA). After reverse transcription, cDNA was fragmented (200–500 bp), purified, end-repaired, adapter-ligated, and processed for strand-specific library construction. rRNA was removed by InDA-C enzymatic digestion. Amplified libraries were purified and quantified using the Qubit dsDNA HS Assay. Library size distribution was assessed using an Agilent 2100 instrument, and libraries with a main peak near 350 bp were sequenced. VIM-AS1 was detected in all three sequenced EV samples.

### 2.14 Plasmid construction and transfection

The lncRNA-VIM-AS1 overexpression plasmid (oe-VIM-AS1) and negative control (oe-NC) were synthesized by GuangZhou Jennio Biotech Co., Ltd. The sh-VIM-AS1 kit, si-Per2 kit, miR-34a-5p mimics, inhibitor NC, and miR-34a-5p inhibitor were purchased from Huzhou Hippo Biotechnology Co., Ltd. Transfection was performed using Lipofectamine 2000 (Invitrogen, Hangzhou, China), and efficiency was verified by qPCR.

### 2.15 *In vitro* detection of lncRNA-VIM-AS1 transfer in a co-culture system

HUCMSCs were transfected with GFP-labeled lncRNA-VIM-AS1 (2 μg/mL) using Lipofectamine 2000 and then co-cultured with BV2 cells. After 24 h, BV2 cells were collected, resuspended in PBS, and analyzed by flow cytometry (BD FACSCALIBUR) to detect VIM-AS1 transfer.

### 2.16 Dual-luciferase reporter gene assay

Candidate interaction sequences were screened using miRDB and StarBase. Cells were co-transfected with wild-type or seed-mutant reporter constructs together with miR-34a-5p mimic or a non-targeting mimic control; Renilla/firefly luciferase ratios were measured 24 h later (Turner BioSystems). Sanger sequencing confirmed that the Per2 reporter contained the mouse 3′UTR context. Cross-species analysis used NCBI reference transcripts NM_022817.3 (human PER2) and NM_011066.4 (mouse Per2) and miRBase mature sequences. Human and mouse miR-34a-5p were identical and the Per2 7mer-A1 seed-matching context was conserved. No annotated mouse ortholog of human VIM-AS1 was identified.

### 2.17 Statistical analysis

Statistical analyses were performed using GraphPad Prism 9.0 and independently audited from the supplied source files. Data are presented as mean ± SEM unless otherwise specified, and n denotes independent biological units. Two-group comparisons used two-sided independent-samples tests; multiple-group comparisons used one-way ANOVA with Tukey adjustment. Neurological scores from the same mice were analyzed by group × time repeated-measures ANOVA with Greenhouse–Geisser correction; prespecified time-point contrasts used Welch tests with Holm adjustment. Survival was analyzed by the Kaplan–Meier method and log-rank test. IBA1 morphometry used animal-level means and exact permutation comparisons because n = 3/group. Exact P values are provided in the source-data tables. A two-sided P < 0.05 was considered statistically significant. No *post hoc* power calculation was used to infer adequacy.

## 3 Results

### 3.1 Characterization of human umbilical cord mesenchymal stem cells and MSC-EVs

The MSC-EV preparation used for functional experiments was isolated by differential ultracentrifugation. In a confirmatory aliquot from the same production batch, TEM showed membrane-bound cup-shaped/concave vesicles, and NTA showed a mean particle diameter of 123.1 nm, a modal diameter of 104.6 nm, and a concentration of 3.6 × 10^10 particles/mL (**Supplementary Figures 1A, B**). CD9, CD63, CD81, and TSG101 were detected in the EV preparation, whereas Calnexin was detected in MSC lysate but not the EV lane (**Supplementary Figure 1C**). The particle-to-protein ratio was 4.99 × 10^10 particles/mg. Together, these results support an MSC small-EV-enriched preparation but do not establish complete biochemical purity or exclude free nucleic acids.

### 3.2 Protective effects of MSC-EV on HS mice and neurological damage

In the independent survival cohort (n = 15/group; animals not shared with the tissue or neurological-score cohorts), 72-h survival increased from 13.3% after HS to 46.7% with MSC-EV treatment; the complete Kaplan–Meier comparison was significant (log-rank χ² [1] = 5.061, P = 0.0245; **Supplementary Figure 2A**). Neurological scores were repeatedly measured in n = 5 mice/group. Across baseline to 72 h, the group × time interaction was significant after Greenhouse–Geisser correction (F[4,32] = 5.444, adjusted P = 0.0103, partial η² = 0.405); a sensitivity analysis restricted to 3–72 h did not show a significant interaction (F[3,24] = 1.884, adjusted P = 0.1787), although the overall treatment-group effect remained significant. Holm-adjusted between-group P values were 0.0239 at 3, 12, and 24 h and 0.00164 at 72 h. HE and Nissl staining showed less hippocampal structural injury and neuronal loss in treated animals (**Supplementary Figures 2B–D**).

### 3.3 MSC-EVs attenuate hippocampal inflammatory responses after heat stroke

In hippocampal homogenates, HS produced time-dependent increases in TNF-α, IL-1β, and IL-6 and altered IL-10 and TGF-β expression (**Supplementary Figure 3**). MSC-EV treatment reduced TNF-α at 24 h, IL-1β at 12–72 h, and IL-6 at 3–24 h, while increasing IL-10 across the measured recovery period and TGF-β at 3–24 h. These tissue-level measurements indicate a shift in the hippocampal inflammatory milieu; because whole hippocampal homogenate rather than isolated microglia was analyzed, they cannot be attributed exclusively to microglia.

### 3.4 MSC-EVs modulate hippocampal microglial phenotype-associated markers

IBA1 immunofluorescence showed greater hippocampal immunoreactivity after HS and lower immunoreactivity after MSC-EV treatment at several time points (**Figure 1A**). IBA1 identifies microglia/macrophages but does not alone establish an activation state. In parallel, hippocampal MCP-1, iNOS, and CD86 increased after HS and were lower with MSC-EV treatment, whereas Arg-1, Fizz1, and CD206 showed the opposite pattern (**Figures 1B–G**). We therefore interpret these findings as treatment-associated changes in inflammatory- and repair-associated marker panels rather than conversion between discrete M1 and M2 states.

**Figure 1:**
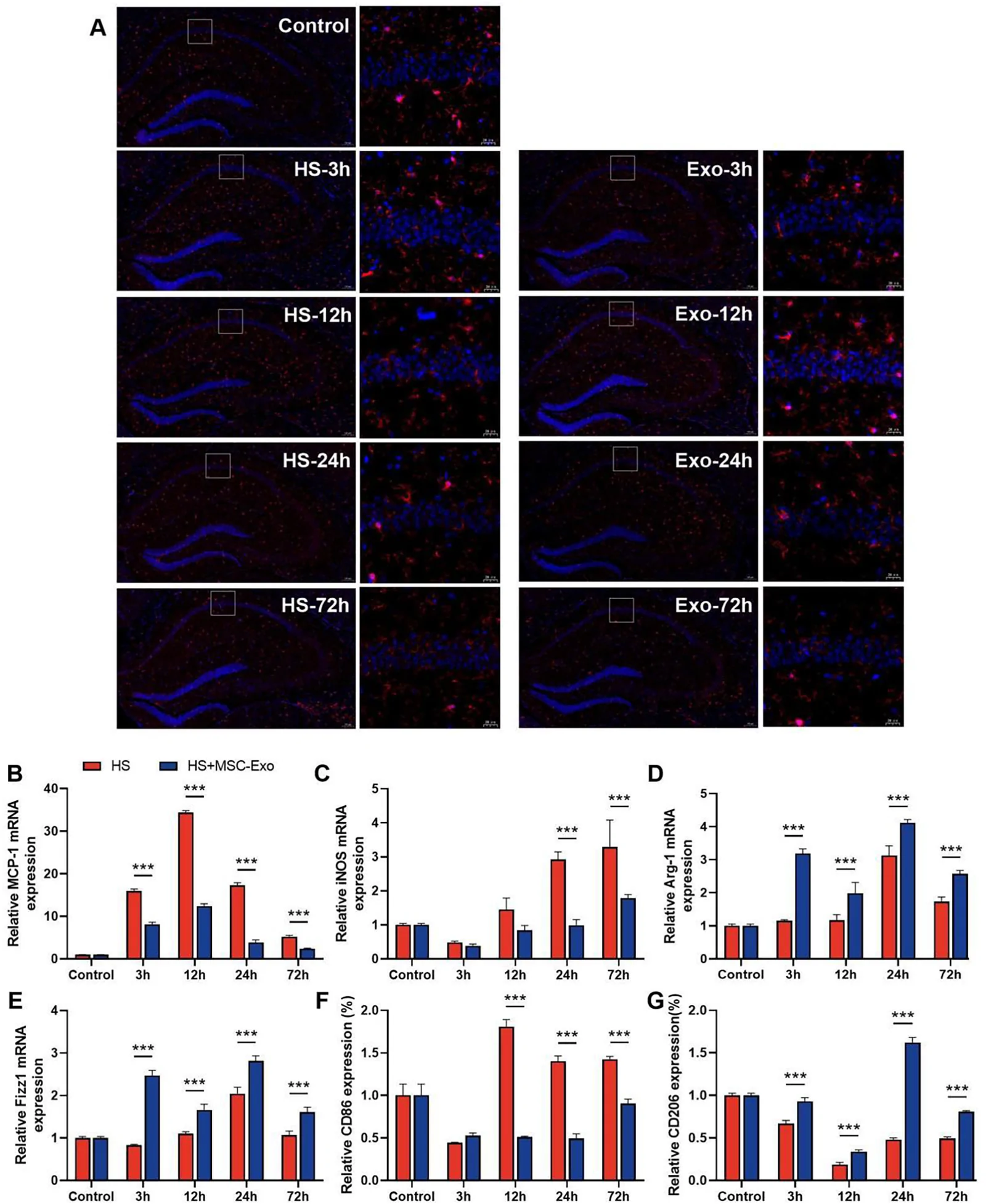
Effects of MSC-EV treatment on hippocampal IBA1 immunoreactivity and phenotype-associated markers after heat stroke. **(A)** Representative hippocampal IBA1 immunofluorescence. **(B–E)** Hippocampal MCP-1, iNOS, Arg-1, and Fizz1 mRNA across recovery times. **(F, G)** CD86- and CD206-positive cell proportions. Data are mean ± SEM; n = 3 independent animals/group/time point. IBA1 indicates immunoreactivity and is not interpreted as activation by itself. Significance levels correspond to the comparisons shown. (***P < 0.001).

### 3.5 MSC co-culture modulates inflammatory readouts in heat-stressed BV2 cells

In the Transwell model, heat-stressed BV2 cells co-cultured with MSCs had lower TNF-α, IL-1β, and IL-6 and higher IL-10 and TGF-β than heat-stressed BV2 cells cultured alone (**Supplementary Figures 4A–E**). MSC co-culture also lowered iNOS, MCP-1, CD45, and CD86 and increased Arg-1, Fizz1, Ym-1, and CD206 at the mRNA, protein, or flow-cytometric level (**Supplementary Figures 4F–M**). These findings show that indirect MSC communication modifies inflammatory readouts in BV2 cells; the Transwell design does not distinguish EVs from other soluble mediators.

### 3.6 VIM-AS1 gain-of-function in MSCs modulates heat-stressed BV2 cells

RNA sequencing detected abundant VIM-AS1 in the MSC-EV RNA preparation across three sequenced samples (**Figure 2A**). VIM-AS1 expression was lower in heat-stressed BV2 cells and higher after MSC co-culture (**Figure 2B**). Fluorescence was detected in BV2 cells after co-culture with MSCs transfected with GFP-labeled VIM-AS1 (**Figure 2C**), supporting transfer of labeled material within the co-culture system but not proving that intact VIM-AS1 was carried exclusively by EVs. Compared with control MSCs, VIM-AS1-overexpressing MSCs produced larger changes in cytokines and inflammatory-/repair-associated markers in heat-stressed BV2 cells (**Figures 2D–G**). Thus, VIM-AS1 gain-of-function in donor MSCs was associated with enhanced anti-inflammatory activity, while purified VIM-AS1-modified EVs were not tested.

**Figure 2:**
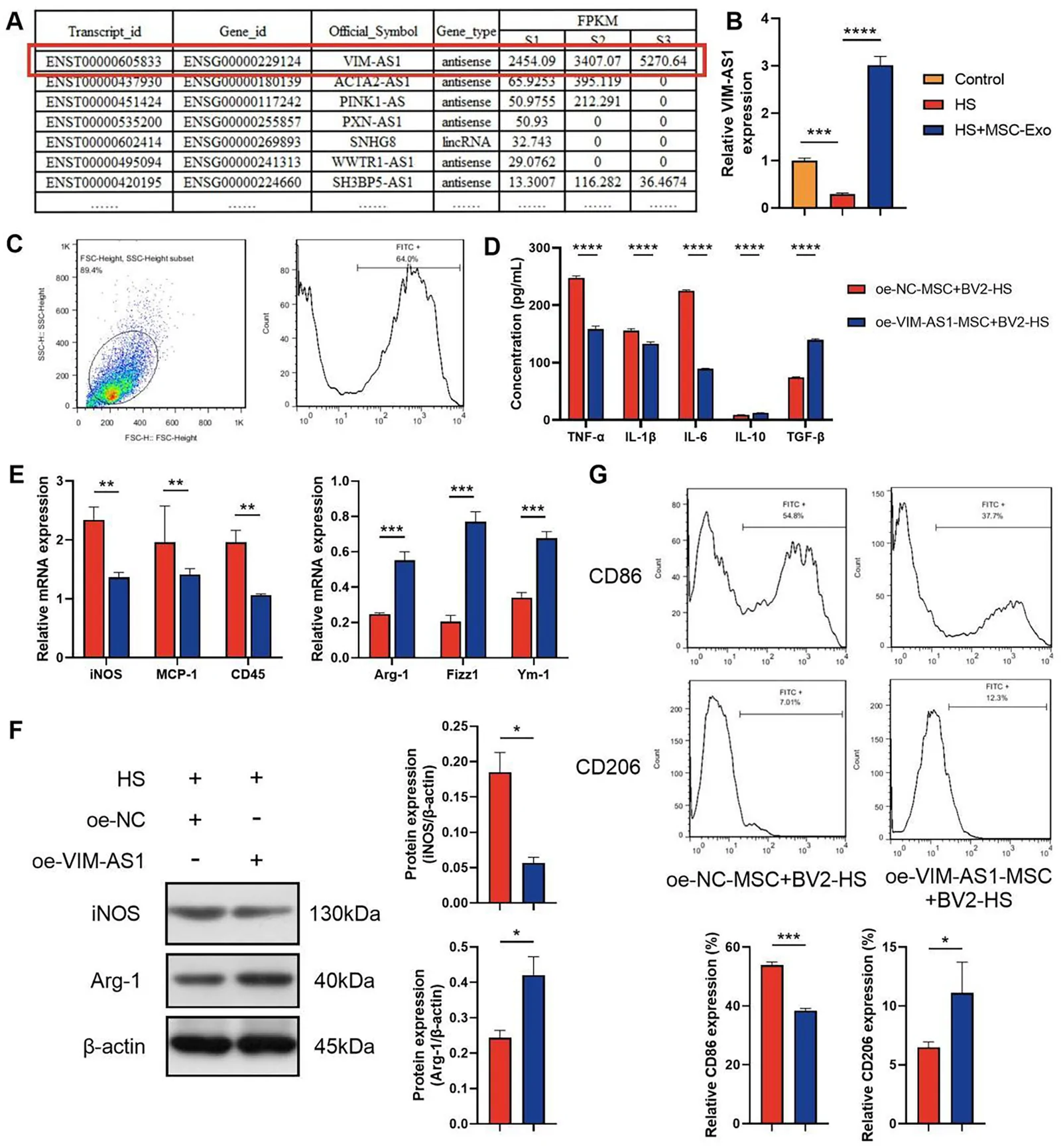
VIM-AS1 gain-of-function in MSCs modifies inflammatory readouts in heat-stressed BV2 cells. **(A)** EV lncRNA-sequencing abundance. **(B)** BV2 VIM-AS1 after heat stress and MSC co-culture. **(C)** Flow-cytometric detection of transferred fluorescent material after co-culture with VIM-AS1-labeled MSCs. **(D)** TNF-α, IL-1β, IL-6, IL-10, and TGF-β. **(E)** iNOS, MCP-1, CD45, Arg-1, Fizz1, and Ym-1 mRNA. **(F)** iNOS and Arg-1 immunoblotting. **(G)** CD86- and CD206-positive proportions. Data are mean ± SEM; n = 3 independent cell-culture experiments/group. Significance levels correspond to the comparisons shown. The Transwell experiment permits EV and soluble-factor exchange and is not interpreted as EV-specific transfer. (*P < 0.05, **P < 0.01, ***P < 0.001, ****P < 0.0001).

### 3.7 VIM-AS1-associated miR-34a-5p/Per2 signaling

Bioinformatic screening identified a candidate miR-34a-5p-matching fragment in the VIM-AS1 genomic region and a conserved seed-matching site in the mouse Per2 3′UTR (**Figure 3A**; **Supplementary Table 5**). miR-34a-5p reduced the activity of the wild-type but not seed-mutant reporter constructs (**Figures 3B, C**). In BV2 cells, VIM-AS1 overexpression lowered miR-34a-5p and increased Per2 mRNA and protein, whereas VIM-AS1 silencing produced reciprocal changes (**Figures 3D, F, G**). miR-34a-5p mimic decreased, and inhibitor increased, Per2 expression (**Figures 3E, H**). The Per2 reporter and expression data support direct miR-34a-5p-dependent repression of mouse Per2. However, sequence auditing showed that the VIM-AS1 reporter fragment is not present in the mature NR_108061.1 transcript used for AAV overexpression; consequently, the reporter assay does not by itself establish direct binding of mature VIM-AS1 to miR-34a-5p.

**Figure 3:**
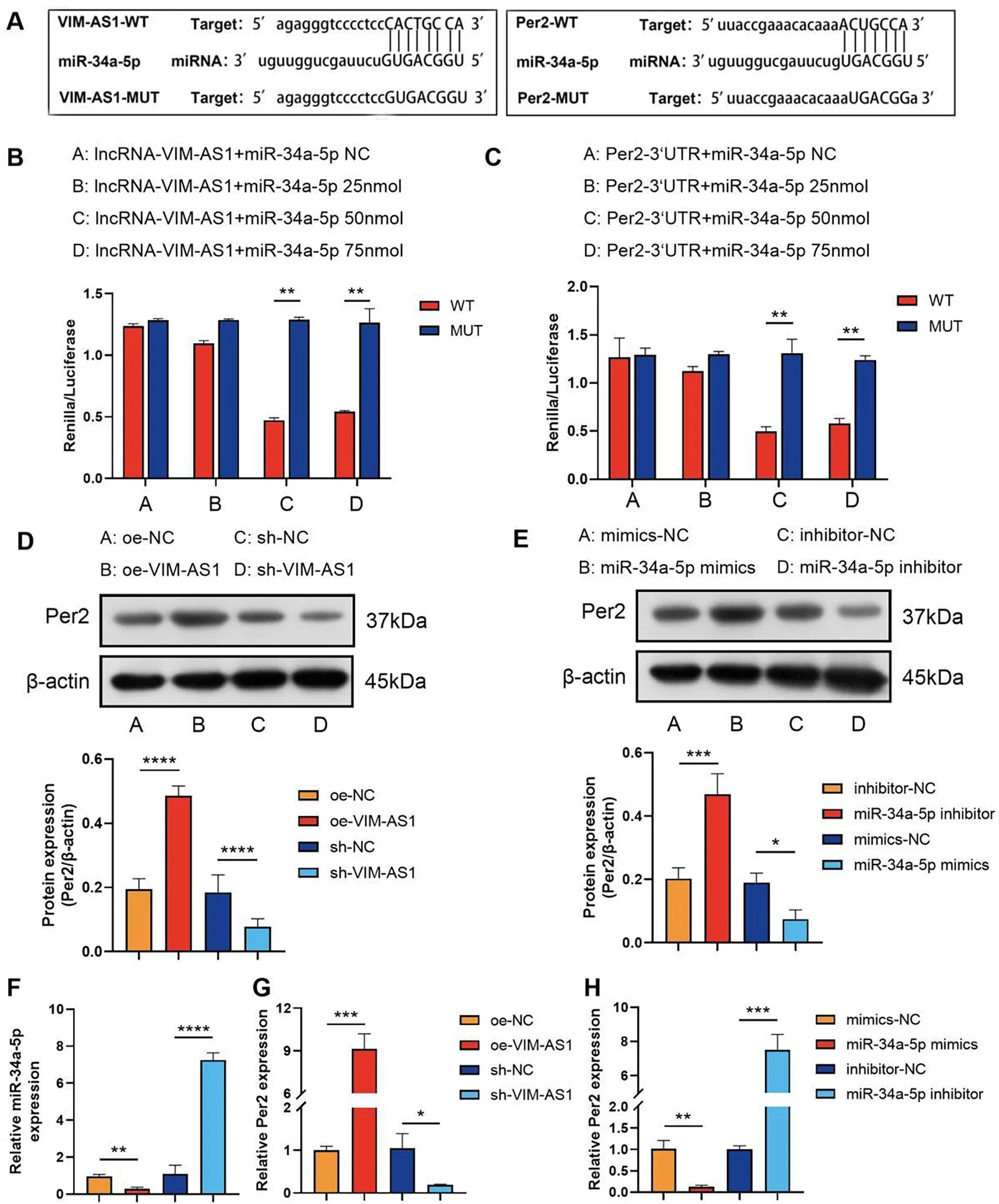
VIM-AS1-associated miR-34a-5p/Per2 signaling. **(A)** Candidate reporter sequences; the Per2 panel has been corrected to the sequenced mouse 3′UTR construct. **(B, C)** Wild-type and seed-mutant reporter responses to miR-34a-5p mimic or non-targeting mimic control. **(D, E)** Per2 immunoblotting after VIM-AS1 or miR-34a-5p perturbation. **(F–H)** miR-34a-5p and Per2 qPCR. Data are mean ± SEM; n = 3 independent experiments/group. Significance levels correspond to the comparisons shown. The VIM-AS1 reporter fragment is not present in the mature NR_108061.1 overexpression transcript and therefore does not alone demonstrate direct mature-VIM-AS1 binding. (*P < 0.05, **P < 0.01, ***P < 0.001, ****P < 0.0001).

### 3.8 Functional roles of miR-34a-5p and Per2 in heat-stressed BV2 cells

Under heat stress, miR-34a-5p mimic or Per2 silencing increased TNF-α, IL-1β, IL-6, iNOS, MCP-1, CD45, and CD86 and reduced IL-10, TGF-β, Arg-1, Fizz1, Ym-1, and CD206 relative to the MSC plus BV2-HS condition (**Figures 4A–I**). The existing combination groups—VIM-AS1-overexpressing MSCs plus miR-34a-5p mimic, and VIM-AS1-overexpressing MSCs plus si-Per2—partially reversed several single-intervention readouts. These data show functional interaction among VIM-AS1 gain-of-function, miR-34a-5p, and Per2-associated perturbations, but they do not establish Per2 as the sole downstream target or replace an *in vivo* reverse-rescue experiment.

**Figure 4:**
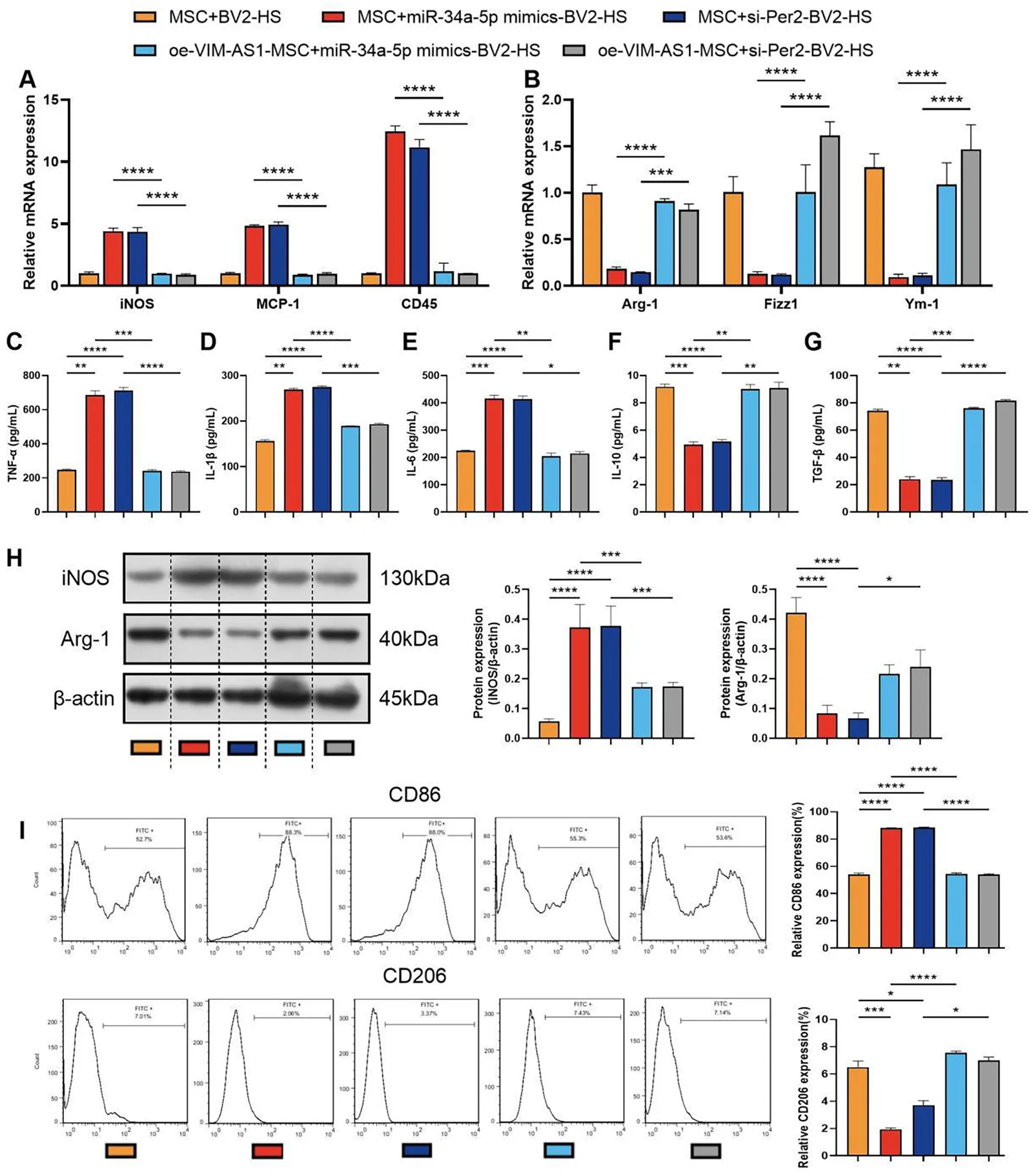
Functional interaction of VIM-AS1 gain-of-function, miR-34a-5p mimic, and Per2 silencing in heat-stressed BV2 cells. Groups are MSC+BV2-HS, MSC+miR-34a-5p mimic+BV2-HS, MSC+si-Per2+BV2-HS, oe-VIM-AS1-MSC+miR-34a-5p mimic+BV2-HS, and oe-VIM-AS1-MSC+si-Per2+BV2-HS. **(A, B)** Inflammatory- and repair-associated mRNA markers. **(C–G)** Secreted cytokines. **(H)** iNOS and Arg-1 immunoblotting. **(I)** CD86- and CD206-positive proportions. Data are mean ± SEM; n = 3 independent experiments/group. Significance levels correspond to the comparisons shown. (*P < 0.05, **P < 0.01, ***P < 0.001, ****P < 0.0001).

### 3.9 AAV-VIM-AS1 modulates hippocampal inflammatory readouts *in vivo*

In the AAV cohort, delivery of human VIM-AS1 increased hippocampal VIM-AS1 and Per2 and decreased miR-34a-5p relative to AAV-vector controls at 3 and/or 24 h after HS (**Figure 5A**; n = 5/group). IBA1-positive area and skeleton-based process complexity were quantified from five fields per mouse (n = 3/group). Relative to vector controls, AAV-VIM-AS1 produced descriptive reductions in IBA1-positive area of 1.04 and 0.37 percentage points at 3 and 24 h, respectively. Process-complexity values were directionally higher with AAV-VIM-AS1 at both times, but none of the exact animal-level permutation comparisons was significant (**Supplementary Figure 6**). Hippocampal inflammatory-associated markers were lower and repair-associated markers higher in the AAV-VIM-AS1 groups (**Figure 5C**; n = 5/group), and neurological scores were improved (**Figure 5D**; n = 5/group). These gain-of-function data support a protective association but do not provide *in vivo* loss-of-function or miR-34a-5p reverse-rescue evidence.

**Figure 5:**
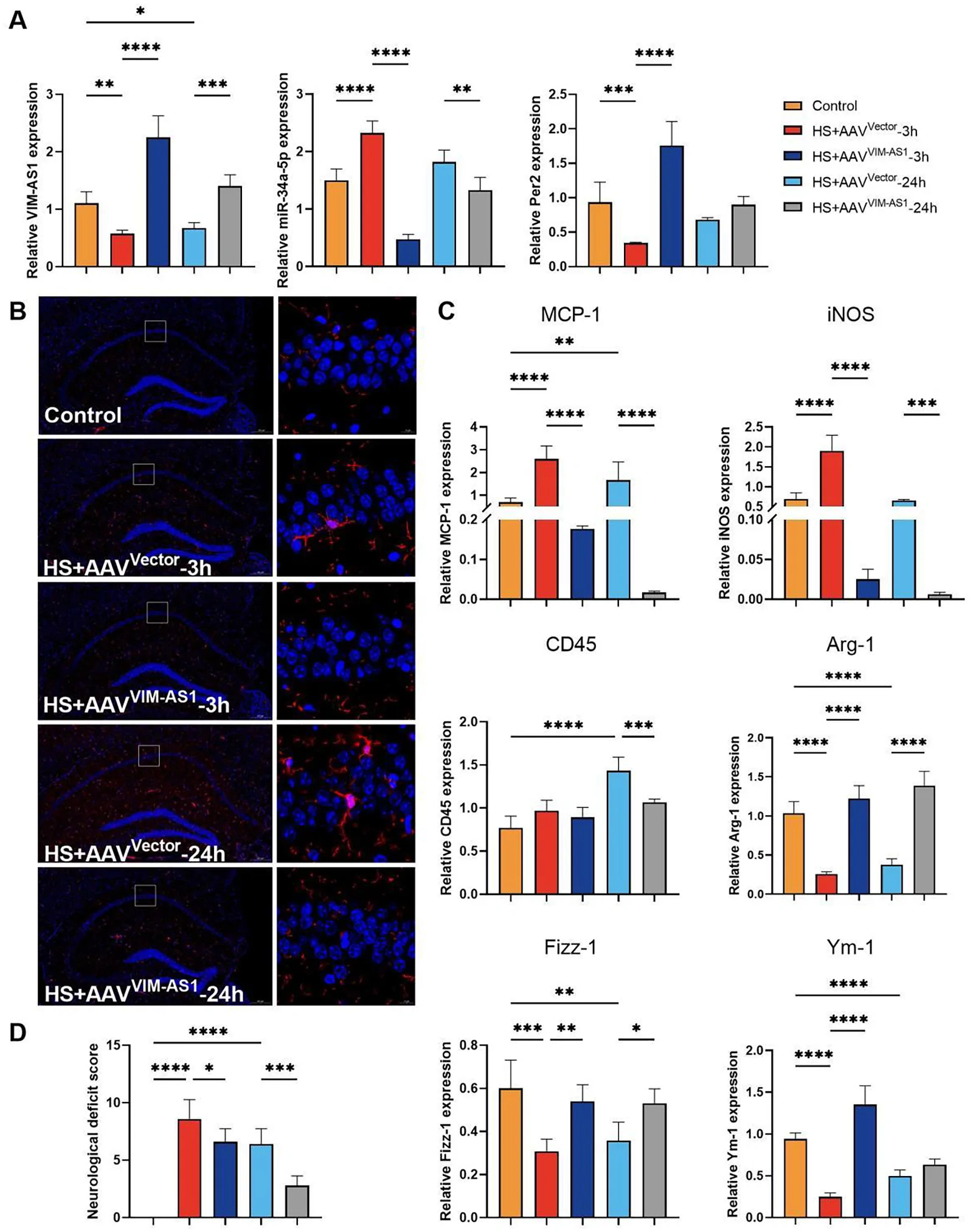
Effects of AAV9-mediated human VIM-AS1 gain-of-function after heat stroke. **(A)** Hippocampal VIM-AS1, miR-34a-5p, and Per2 qPCR in Control, HS+AAV-Vector-3 h, HS+AAV-VIM-AS1–3 h, HS+AAV-Vector-24 h, and HS+AAV-VIM-AS1–24 h groups (n = 5 animals/group). **(B)** Representative hippocampal IBA1 immunofluorescence (n = 3 animals/group; five fields averaged per animal); quantitative morphometry is shown in **Supplementary Figure 6**. **(C)** Hippocampal inflammatory- and repair-associated markers (n = 5 animals/group). **(D)** Neurological deficit scores (n = 5 animals/group). Data are mean ± SEM. Significance levels correspond to the comparisons shown. (*P < 0.05, **P < 0.01, ***P < 0.001, ****P < 0.0001).

## 4 Discussion

Using mouse and BV2 models, we found that MSC-EV treatment improved survival and neurological outcomes after HS and shifted hippocampal and cellular inflammatory readouts toward a less inflammatory profile. The study extends our previous viable-MSC work by testing a cell-free EV preparation and by identifying VIM-AS1-associated changes in miR-34a-5p and Per2. The data support direct repression of mouse Per2 by miR-34a-5p and a protective effect of VIM-AS1 gain-of-function. They do not demonstrate that VIM-AS1 is the sole EV effector or that mature EV VIM-AS1 directly binds miR-34a-5p; these distinctions define the strength and limits of the mechanistic model. More broadly, neuroprotective interventions can converge on oxidative stress, neuroinflammation, apoptosis, mitochondrial dysfunction, and cerebral perfusion (25). Botanical extracts have also promoted rat neural stem-cell proliferation and reduced toxin-associated neuronal death *in vitro* (26). Although these studies do not test HS or MSC-EVs, they place the present findings within a wider context of multimodal neuroprotection.

HS progresses rapidly and carries high mortality, yet its low incidence limits awareness and research. Appropriate animal models are therefore essential. Our team previously developed an improved stepwise heating protocol that closely reproduces the pathophysiological features of clinical HS, including organ injury and systemic inflammation (27).

During HS, thermal stress induces lipid peroxidation and oxidative stress in brain cells, directly damaging the CNS and provoking severe neuroinflammation (28, 29). Previous studies have identified NLRP3/IL-1β-mediated neuroinflammation as a key pathological mechanism in HS, with IL-1β serving as both a severity marker and therapeutic target (7). Our earlier work also showed that HS increases inflammatory cytokines such as IL-1β, IL-6, and TNF-α, as well as chemokines including monocyte chemoattractant protein-1 (MCP-1) and regulated on activation, normal T cell expressed and secreted (RANTES), thereby aggravating neuroinflammation and neuronal injury (22). This may help explain the persistent CNS dysfunction seen in more than 30% of HS survivors (30). Several interventions, including berberine (31), dexmedetomidine (32), and hyperbaric oxygen therapy (33), have shown neuroprotective potential in HS, and our previous studies confirmed similar effects of MSCs (22, 23). Building on this, the present study showed that MSC-EV significantly decreased pro-inflammatory cytokines such as IL-1β while increasing anti-inflammatory cytokines such as IL-10 in brain tissue. MSC-EV also ameliorated hippocampal injury, preserved neuronal survival, and improved neurological function in HS mice, supporting its potential as a therapeutic strategy for HS-related CNS injury.

Microglial responses are heterogeneous and evolve across disease stage, region, and local cues (11–13). Accordingly, iNOS/CD86 and Arg-1/CD206 should be interpreted as limited inflammatory- and repair-associated readouts, not as mutually exclusive M1/M2 identities. In HS models, microglia increase inflammatory mediators and can propagate neuronal apoptosis and autophagy-related injury (28, 32, 34, 35). Our tissue and BV2 data consistently showed lower inflammatory-associated markers and higher repair-associated markers after MSC-EV treatment or MSC co-culture. The added IBA1 morphometry supports a directional change in immunoreactivity, but IBA1 alone is not an activation marker and no phagocytosis assay was performed. Single-cell or spatial profiling and functional phagocytosis assays will be needed to define the responding microglial states.

EV cargo includes many ncRNAs, proteins, and lipids with potentially convergent or opposing functions (36, 37). RNA sequencing identified VIM-AS1 as an abundant lncRNA in the analyzed MSC-EV samples, and VIM-AS1 gain- and loss-of-function altered inflammatory readouts in BV2 cells. AAV-mediated human VIM-AS1 delivery also improved several *in vivo* endpoints. These findings identify VIM-AS1 as a candidate contributor, but not necessarily the dominant EV cargo. More generally, work on co-assembled natural-compound nanocomplexes illustrates that delivery platforms may enhance bioavailability and biocompatibility while still requiring rigorous compositional and physicochemical characterization (38). Biomimetic macrophage-membrane-coated nanoparticles likewise illustrate how a biological interface may improve immune compatibility and targeting, while introducing separate questions of composition, biodistribution, safety, and manufacturing (39). These are conceptual delivery-science comparisons rather than evidence of mechanistic equivalence to MSC-EVs. Furthermore, the donor cells were human whereas BV2 cells and mice were murine. Human and mouse miR-34a-5p are identical and the relevant Per2 seed context is conserved, but our NCBI/Ensembl audit identified no annotated one-to-one mouse ortholog of human VIM-AS1. Because lncRNA transcript structure and primary sequence may be poorly conserved, lack of an annotated ortholog is biologically plausible; nevertheless, it precludes inference of an endogenous conserved mouse VIM-AS1 pathway. The *in vivo* experiment therefore tests ectopic/pharmacological delivery of a human lncRNA rather than modulation of an endogenous conserved mouse transcript.

miR-34a-5p can amplify innate immune signaling and inflammatory cytokine production (40–43). In our experiments, miR-34a-5p mimic worsened inflammatory readouts, whereas inhibition increased Per2. The conserved mouse Per2 3′UTR reporter supports Per2 as a direct miR-34a-5p target. VIM-AS1 perturbation produced reciprocal changes in miR-34a-5p and Per2, and combination experiments showed functional interaction. However, the assayed VIM-AS1 reporter fragment is absent from the mature NR_108061.1 transcript used for overexpression. We therefore refrain from claiming a validated mature-VIM-AS1 ceRNA interaction unless a site-containing alternative transcript is demonstrated by RNA-seq junction evidence or targeted RT-PCR. The current evidence supports VIM-AS1-associated regulation of the miR-34a-5p/Per2 module rather than a fully resolved ceRNA mechanism.

Per2 links circadian timing with innate immune function (44–46). In BV2 cells, Per2 silencing shifted cytokines and phenotype-associated markers toward a more inflammatory profile, supporting a protective association under heat stress. Although Per2 has been linked to PTEN/PI3K/Akt signaling in chondrocytes (47) and to NF-κB/p65-dependent transcription in macrophages (48), neither pathway was tested here. We therefore removed the previous graphical abstract, which could imply an untested Per2–PTEN–PI3K/Akt mechanism, and do not infer this downstream pathway from the present data. Future studies should examine circadian phase, time-of-day effects, and Per2-dependent transcription in primary microglia during HS.

Several limitations should be considered. First, the EV intervention used a complex small-EV-enriched preparation; free-nucleic-acid contamination was not directly measured, the particle-to-protein ratio was modest, and batch-to-batch VIM-AS1 abundance was not established. Second, the Transwell and AAV experiments do not prove that natural MSC-EV protection is mediated mainly by VIM-AS1, and purified EVs from VIM-AS1-silenced or -overexpressing MSCs were not tested. Third, mechanistic experiments relied largely on BV2 cells; primary or acutely isolated microglia, single-cell state mapping, and phagocytosis assays were not available. Fourth, brain qPCR used hippocampal homogenate rather than isolated microglia, and IBA1 alone is not state-specific. Fifth, human VIM-AS1 has no annotated mouse ortholog, limiting cross-species interpretation. Sixth, no hyperthermia-only/equal-thermal-load cohort, *in vivo* AAV loss-of-function, or miR-34a-5p reverse-rescue cohort was included. Seventh, randomization records and technical-replicate metadata should be prospectively standardized in confirmatory studies. These limitations require cautious interpretation and independent replication before clinical translation.

## 5 Conclusions

MSC-EV treatment attenuated hippocampal inflammation and injury and improved survival and neurological outcomes after experimental heat stroke. VIM-AS1 gain-of-function was associated with reciprocal miR-34a-5p/Per2 changes and improved inflammatory readouts, identifying a candidate signaling module for further study. The data do not establish VIM-AS1 as the sole EV effector or a fully validated mature-transcript ceRNA, and confirmatory studies using VIM-AS1-modified purified EVs and primary microglia are required.

## Data Availability

The data presented in the study are deposited in the Figshare repository, accession number https://doi.org/10.6084/m9.figshare.33421912.
