# Supplementary material for: Mesenchymal stem cell-derived extracellular vesicles ameliorate heat stroke-induced hippocampal injury and modulate VIM-AS1-associated miR-34a-5p/Per2 signaling
Source: Front Immunol. 2026 Sep 14;17:1873819. doi: 10.3389/fimmu.2026.1873819 (PMC13616672; doi:10.3389/fimmu.2026.1873819)
Supplement: Supplementary file 1 [file SupplementaryFile1.docx]

# Supplementary Materials

Mesenchymal Stem Cell-Derived Extracellular Vesicles Ameliorate Heat Stroke-Induced Hippocampal Injury and Modulate VIM-AS1-Associated miR-34a-5p/Per2 Signaling

Lu Wang1, 2, *, Min Wang3, 4, *, Yuyan Liu5, *, Yang Bai6, Yun Li7, Yuan Cao3, 4, Chengjin Wang3, 4, Zhen Gao3, 4, Yiqi Wu3, 4, Zihui Deng8, #, Hongjun Kang2,4,#

1.Department of Critical Care Medicine, the Fifth Medical Centre, Chinese PLA General Hospital, Beijing 100853, China;

2. National Key Laboratory of Kidney Diseases, Beijing Key Laboratory of Kidney Disease Research, National Clinical Research Center for Kidney Diseases, Beijing 100853, China;

3. Medical School of Chinese PLA, Beijing 100853, China;

4. Department of Critical Care Medicine, the First Medical Centre, Chinese PLA General Hospital, Beijing 100853, China;

5. Department of Critical Care Medicine, Beijing Armed Police Corps Hospital, Beijing 100027, China;

6. Department of Emergency Medicine, the First Medical Centre, Chinese PLA General Hospital, Beijing 100853, China;

7. Department of Critical Care Medicine, PLA General Hospital of Southern Theatre Command, Guangzhou 510010, China;

8. Department of Basic Medicine, Medical School of Chinese PLA, Beijing 100853, China.

*Lu Wang, Min Wang and Yuyan Liu contributed equally to this work.

#Correspondence:

Zihui Deng

dzihui123@163.com

Hongjun Kang

doctorkang301@163.com

Department of Critical Care Medicine, the First Medical Centre, Chinese PLA General Hospital,

No. 28, Fuxing Road, Haidian District, Beijing 100853, China.

## Supplementary Figure S1. Characterization of the MSC-EV preparation.

(A) Transmission electron micrographs at the indicated scale bars. (B) Nanoparticle tracking analysis. The mean diameter was 123.1 nm, the modal diameter was 104.6 nm, and the particle concentration was 3.6 × 10^10 particles/mL. (C) Immunoblotting of MSC lysate (Cell) and the EV preparation (EXOs) for Calnexin, CD63, TSG101, CD9, and CD81. Calnexin was detected in MSC lysate but not in the EV lane. The particle-to-protein ratio was 4.99 × 10^10 particles/mg. The confirmatory analyses used an archived aliquot from the same production batch as the functional-study preparation.

## Supplementary Figure S2. Survival, neurological scores, and hippocampal injury after MSC-EV treatment.

(A) Kaplan–Meier survival curves from an independent cohort (n = 15 mice/group); log-rank χ²(1) = 5.061, P = 0.0245. These animals were not used for tissue, molecular, histological, or neurological-score endpoints. Numbers at risk for HS versus HS+MSC-EV at 0, 6, 12, 18, 24, 36, 42, 48, and 72 h were 15/15, 15/15, 14/15, 9/14, 6/11, 5/9, 4/8, 3/8, and 2/7, respectively. (B) Neurological deficit scores repeatedly measured in the same mice (n = 5/group). The baseline-to-72-h group × time interaction was significant after Greenhouse–Geisser correction (F[4,32] = 5.444, adjusted P = 0.0103, partial η² = 0.405). Holm-adjusted between-group P values were 0.0239 at 3, 12, and 24 h and 0.00164 at 72 h. (C) Representative hippocampal hematoxylin–eosin staining and pathological injury scores. (D) Representative Nissl staining and surviving-neuron counts. Histological quantification used n = 3 independent animals/group/time point. Data are mean ± SEM. Scale bars, 50 μm.

## Supplementary Figure S3. Hippocampal inflammatory-mediator time courses.

Hippocampal mRNA levels of (A) TNF-α, (B) IL-1β, (C) IL-6, (D) IL-10, and (E) TGF-β after heat stroke with or without MSC-EV treatment. Measurements were obtained from hippocampal homogenates rather than isolated microglia. Data are mean ± SEM; n = 3 independent animals/group/time point. *P < 0.05, **P < 0.01, ***P < 0.001, and ****P < 0.0001 for the comparisons shown.

## Supplementary Figure S4. Initial BV2 heat-stress and MSC indirect co-culture experiments.

(A–E) TNF-α, IL-1β, IL-6, IL-10, and TGF-β in culture supernatants. (F,H,I) iNOS and Arg-1 immunoblotting and densitometry. (G,J,K) CD86- and CD206-positive proportions. (L–Q) iNOS, MCP-1, CD45, Arg-1, Fizz1, and Ym-1 mRNA. The 0.4-μm Transwell system permits exchange of EVs and soluble factors and is not interpreted as proof of EV-specific transfer. Data are mean ± SEM; n = 3 independent cell-culture experiments/group. *P < 0.05, **P < 0.01, ***P < 0.001, and ****P < 0.0001 for the comparisons shown.

## Supplementary Figure S6. Animal-level IBA1 morphometry in the AAV cohort.

(A) IBA1-positive area. (B) Skeleton-based process-complexity index. Five hippocampal fields were averaged per mouse; n = 3 independent animals/group. Bars show mean ± SEM. A single global red-channel-specific threshold was applied to all images. The process-complexity index is an image-derived morphology proxy and not a phagocytosis assay. Exact animal-level permutation comparisons were not significant; therefore, no significance symbols are shown.

## Supplementary Table S5. Cross-species sequence and annotation audit

| **Item** | **Human** | **Mouse** | **Interpretation/source** |
| --- | --- | --- | --- |
| Mature miR-34a-5p | UGGCAGUGUCUUAGCUGGUUGU | UGGCAGUGUCUUAGCUGGUUGU | Identical 22-nt mature sequences; miRBase MIMAT0000255/MIMAT0000542 |
| PER2/Per2 reporter context | TTACCGAAACACAAAACTGCCA | TTACTTAAACACAAAACTGCCA | 20/22 nt identity; conserved AACTGCC 7mer-A1 context; NM_022817.3/NM_011066.4 |
| VIM-AS1 orthology | Annotated human lncRNA | No annotated one-to-one mouse ortholog identified | Mouse AAV work tests delivered human RNA, not an endogenous conserved mouse transcript |
| VIM-AS1 reporter fragment | AGAGGGTCCCCTCCCACTGCCA | Not applicable | The fragment is absent from mature NR_108061.1 used for AAV overexpression; the reporter does not alone validate direct mature-transcript binding |

Table numbering is retained as S5 to remain consistent with the point-by-point reviewer response and manuscript citations.
